# Risk predictive value of white blood cell count in patients with diabetes‑associated lower urinary tract symptoms: A population-based study

**DOI:** 10.1016/j.clinsp.2026.100928

**Published:** 2026-04-17

**Authors:** Ning Liu, Dingjie Zhou, Nihao Cao

**Affiliations:** a Department of Urology, Affiliated Zhongda Hospital of Southeast University, Nanjing, China; b Jiangsu Health Development Research Center, NHC Contraceptives Adverse Reaction Surveillance Center, Jiangsu Provincial Medical Key Laboratory of Fertility Protection and Health Technology Assessment, Nanjing, China; c Department of Urology, Nantong Haimen People's Hospital, Nantong, China

**Keywords:** Diabetes‑associated lower urinary tract symptoms, White blood cell count, National health and nutrition examination survey

## Abstract

**Background:**

The prevalence of diabetes‑associated Lower Urinary Tract Symptoms (LUTS) is relatively low, and there are limited clinical studies on its associated factors. The present study proposed to investigate the predictive value of various inflammatory indices in patients with diabetes‑associated LUTS.

**Methods:**

Utilizing data from the National Health and Nutrition Examination Survey 2005‒2008, patients were categorized into four groups: normal group, Diabetes Mellitus (DM) group, LUTS group, and diabetes‑associated LUTS group. Inflammatory indices included C-Reactive Protein (CRP), White Blood Cell (WBC) count, Neutrophil-to-Lymphocyte Ratio (NLR), Platelet-to-Lymphocyte Ratio (PLR), Systemic Inflammation Response Index (SIRI), and Systemic Inflammation Index (SII). Multivariate logistic regression and Restricted Cubic Splines (RCS) were employed to evaluate the relationship between inflammatory indices and diabetes‑associated LUTS.

**Results:**

A total of 5680 participants were included in the final analysis, comprising 3779 females and 1901 males. The prevalence of diabetes‑associated LUTS was 6.9% in females and 2.2% in males. Diabetes-associated LUTS was associated with older age, lower educational level, hypertension, congestive heart failure, coronary heart disease, emphysema, and higher body mass index and inflammatory indices. Furthermore, multivariate logistic regression analysis revealed that WBC count was a significant factor for both female and male diabetes‑associated LUTS patients, with higher WBC levels correlating with increased diabetes‑associated LUTS risk (females: adjusted Odds Ratio [OR] = 1.115 [1.032‒1.205], *p* = 0.006; males: OR = 1.207 [1.083‒1.345], *p* = 0.001). RCS revealed a positive association between WBC count and diabetes‑associated LUTS in female and male participants.

**Conclusion:**

Inflammatory markers such as CRP and WBC count were higher in the diabetes‑associated LUTS group compared to the other three groups. WBC count is an associated factor for diabetes‑associated LUTS, and incorporating WBC count into clinical assessments can aid in identifying diabetes‑associated LUTS patients, thereby facilitating targeted interventions.

## Highlights

• Diabetes‑associated LUTS were associated with higher BMI and inflammatory indices.
• CRP and WBC count were higher in the diabetes‑associated LUTS group.
• WBC count was an independent risk factor in diabetes‑associated LUTS patients.

## Introduction

Diabetes Mellitus (DM) is a chronic metabolic disorder characterized by persistently elevated blood glucose levels.^1^ Prolonged hyperglycemia can lead to complications such as cardiovascular diseases, kidney disease, bladder dysfunction, nerve damage, and retinopathy.^2^ Diabetic patients often exhibit urodynamic abnormalities, including reduced bladder sensation, increased post-void residual urine volume, and decreased detrusor muscle contractility.^3^ Diabetes‑associated Lower Urinary Tract Symptoms (LUTS), a common chronic complication in diabetic patients, manifests clinically as urinary frequency, urgency, incomplete bladder emptying, difficulty urinating, and urinary retention, significantly impacting quality of life.^4^ Diabetes-induced bladder dysfunction, termed diabetic cystopathy, is classically characterized by impaired bladder sensation, increased bladder capacity, reduced detrusor contractility, and elevated post‑void residua.^5^ These features are defined through urodynamic testing and/or objective measures of bladder emptying. Diabetes‑associated LUTS is characterized by progressive impairment of bladder sensation, diminished detrusor muscle contractility, increased post-void residual urine volume, and an elevated risk of urinary tract infections.^6^ Its pathophysiological mechanisms are complex, involving multiple intertwined processes such as nerve damage, microvascular dysfunction, metabolic disturbances, smooth muscle dysfunction, chronic inflammation/oxidative stress, and structural remodeling.^7^^,^^8^

Extensive research highlights the critical roles of oxidative stress (Reactive Oxygen Species, ROS) and inflammatory responses in diabetes pathogenesis.^9^ ROS induces oxidative tissue damage and promotes the aggregation of inflammatory cells, triggering the release of associated inflammatory cytokines and growth factors.^10^ Infiltration of inflammatory cells (e.g., lymphocytes and neutrophils) further recruits and releases various inflammatory cytokines, which in turn regulate ROS levels.^11^ Inflammatory markers such as C-Reactive Protein (CRP), white blood cell count, Neutrophil-to-Lymphocyte Ratio (NLR), Platelet-to-Lymphocyte Ratio (PLR), Systemic Inflammatory Response Index (SIRI), and Systemic Immune-Inflammation Index (SII) have emerged as potential indicators of systemic inflammation in DM-related conditions.^12^, ^13^, ^14^, ^15^ However, the utility of these markers in diabetes‑associated LUTS patients remains unclear. In this study, we analyzed clinical data from diabetes‑associated LUTS patients in the 2005–2008 National Health and Nutrition Examination Survey (NHANES) database to evaluate the applicability of these inflammatory biomarkers in diabetes‑associated LUTS patients.

## Materials and methods

### Data source and study population

The NHANES is a cross-sectional survey conducted by the National Center for Health Statistics (NCHS) in the United States to assess the health and nutritional status of American adults and children.^16^ The NHANES database includes demographic information, socioeconomic data, dietary status, health-related questions, as well as medical examinations and laboratory tests related to health. NHANES is a publicly available database, and since 1999, data files of the survey conducted every two years have been released online (https://wwwn.cdc.gov/nchs/nhanes/default.aspx). The current study includes NHANES public data from two cycles between 2005 and 2008, encompassing a total of 20,497 participants. This study follows the STROBE Statement. This study used previously collected deidentified data, which was deemed exempt from review by the Ethics Committee of the Affiliated Zhongda Hospital of Southeast University (2024ZDSYLL458-P01).

### Definition and diagnostic criteria

In this study, diabetes‑associated LUTS were defined as DM combined with clinical symptoms of LUTS.^17^ The diagnosis of LUTS was based on specific NHANES questionnaire items:

Urinary hesitancy: Diagnosed using the KIQ081 question: “Do you usually have trouble starting to urinate?” Participants answering “yes” were classified as having urinary hesitancy.

Incomplete emptying: Diagnosed using the KIQ101 question: “After urinating, does your bladder feel empty?” Participants answering “no” were classified as having incomplete emptying.

Urinary Frequency: Diagnosed using the KIQ005 question: “How often do you usually have to urinate immediately after you feel the need to urinate?” Participants answering “frequently” were classified as having urinary frequency.

Nocturia: Diagnosed using the KIQ480 question: “During the past 30-days, how many times per night did you most typically get up to urinate, from the time you went to bed at night until the time you got up in the morning?” Participants reporting two or more episodes per night were classified as having nocturia.

Since female participants in the database only answered KIQ005 and KIQ480, the diagnostic criteria for clinical LUTS were adjusted accordingly. Male participants with two or more symptoms (urinary hesitancy, incomplete emptying, urinary frequency, and nocturia) are defined as having clinical LUTS symptoms, while female participants with urinary frequency or nocturia are defined as having clinical LUTS symptoms.

### Exclusion criteria

**Fig. 1:**
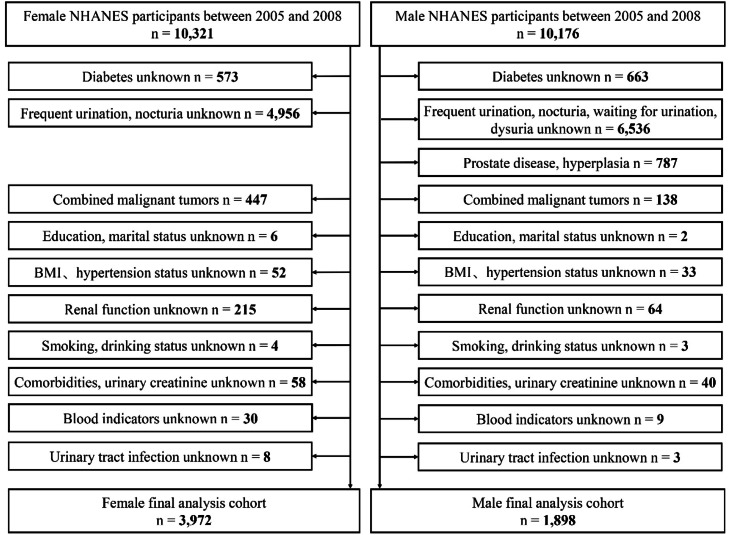
Flowchart for exclusion of data.

The exclusion of urinary tract infections was determined based on the ICD-10-CM codes in the Prescription Medications questionnaire. For patients responding with N39.0 and N39.0P, they were considered to have comorbid urinary tract infections. Given the differing diagnostic criteria for diabetic bladder dysfunction between male and female patients, separate exclusion criteria were established for each group (Fig. 1). Normal group: participants without DM and LUTS; LUTS group: participants with LUTS but without DM; DM group: participants with DM without LUTS; diabetes‑associated LUTS group: participants with DM combined with LUTS. Finally, a total of 3980 male and 1901 female participants were included in the final study.

### Research variables

In this study, the main research variables include the following: gender (male, female), age (≤ 50-years-old, > 50-years-old), race (Mexican American, other Hispanic, non-Hispanic white, non-Hispanic black, other races), education level (less than high school, high school, college or above), marital status (married, unmarried), body mass index (BMI < 25 kg/m^2^, 25‒29.9 kg/m^2^, ≥ 30 kg/m^2^), smoking (never, former, current), alcohol consumption (yes, no), hypertension (yes, no), congestive heart failure (yes, no), coronary heart disease (yes, no), pulmonary emphysema (yes, no), chronic bronchitis (yes, no), metformin use (yes, no), HbA1c (%), CRP (mg/dL), white blood cell count (WBC, 1000 cells/uL), lymphocyte count (1000 cells/uL), monocyte count (1000 cells/uL), platelet count (1000 cells/uL), and neutrophil count (1000 cells/uL). The NLR was calculated as the neutrophil count/lymphocyte count; the PLR is the platelet count/lymphocyte count; the SII is the platelet count × neutrophil count/lymphocyte count; the SIRI is the monocyte count × neutrophil count/lymphocyte count.

### Statistical methods

In our study, the distribution of continuous variables was described using mean ± standard deviation, while the distribution of categorical variables was calculated using proportions (%). Analysis of variance (ANOVA) was used to assess the differences between the different groups. Multifactor logistic regression analysis was used to assess the relationship between different inflammatory indices and diabetes‑associated LUTS, with results expressed as Odds Ratios (OR) and 95% Confidence Intervals (95% CI). Receiver Operating Characteristic (ROC) curves and dose–response curves of Restricted Cubic Spline (RCS) were used to assess the relationship between WBC and diabetes‑associated LUTS. Statistical analysis in this study was conducted using SPSS (22.0) software, GraphPad Prism 8.0 software, and R software (version 3.5.3) were used for plotting. All statistical analyses were two-tailed tests, with a significance level set at *p* < 0.05.

## Results

### Comparison of general condition among female participants

Among 3980 female participants, 6.9% (275) were diagnosed with diabetes‑associated LUTS, 3.5% (139) with DM, and 39.7% (1581) with LUTS. The clinical baseline data of the four groups are compared in Table 1. Significant differences were observed among the four groups in terms of age, race, education level, BMI, alcohol consumption, hypertension, congestive heart failure, coronary heart disease, chronic bronchitis, and metformin use. The diabetes‑associated LUTS group had a higher proportion of non-Hispanic Blacks, lower education levels, BMI ≥ 30 kg/m^2^, hypertension, congestive heart failure, and coronary heart disease compared to the other three groups.

**Table 1:** Clinical baseline characteristics of the 3980 female participants between 2005 and 2008.

Variables	Total patients	Normal	DM	LUTS	Diabetes‑associated LUTS	p
	n (%)	n (%)	n (%)	n (%)	n (%)	
Total patients	3972	1981 (49.9)	137 (3.4)	1580 (39.8)	274 (6.9)	
Age, years						<0.001
≤ 50	2334 (58.8)	1421 (71.7)	31 (22.6)	819 (51.8)	63 (23.0)	
> 50	1638 (41.2)	560 (28.3)	106 (77.4)	761 (48.2)	211 (77.0)	
Race						<0.001
Mexican Americans	820 (20.6)	391 (19.7)	32 (23.4)	335 (21.2)	62 (22.6)	
Other Hispanics	334 (8.4)	169 (8.5)	14 (10.2)	125 (7.9)	26 (9.5)	
Non-Hispanic whites	1838 (46.3)	977 (49.3)	43 (31.4)	726 (45.9)	92 (33.6)	
Non-Hispanic blacks	828 (20.8)	361 (18.2)	42 (30.7)	337 (21.3)	88 (32.1)	
Other races	152 (3.8)	83 (4.2)	6 (4.4)	57 (3.6)	6 (2.2)	
Marital status						0.253
Married	1998 (50.3)	1014 (51.2)	60 (43.8)	795 (50.3)	129 (47.1)	
Unmarried	1974 (49.7)	967 (48.8)	77 (56.2)	785 (49.7)	145 (52.9)	
Educational level						<0.001
Less than high school	1085 (27.3)	415 (20.9)	43 (31.4)	505 (32.0)	122 (44.5)	
High school	924 (23.3)	438 (22.1)	47 (34.3)	375 (23.7)	64 (23.4)	
College or above	1963 (49.4)	1128 (56.9)	47 (34.3)	700 (44.3)	88 (32.1)	
BMI, kg/m^2^						<0.001
< 25	1254 (31.6)	792 (40.0)	18 (13.1)	409 (25.9)	35 (12.8)	
25‒29.9	1175 (29.6)	587 (29.6)	35 (25.5)	502 (31.8)	51 (18.6)	
≥ 30	1543 (38.8)	602 (30.4)	84 (61.3)	669 (42.3)	188 (68.6)	
Smoking						0.073
Never	2476 (62.3)	1255 (63.4)	83 (60.6)	963 (60.9)	175 (63.9)	
Former	761 (19.2)	344 (17.4)	32 (23.4)	326 (20.6)	59 (21.5)	
Current	735 (18.5)	382 (19.3)	22 (16.1)	291 (18.4)	40 (14.6)	
Alcohol consumption						<0.001
Yes	2344 (59.0)	1264 (63.8)	60 (43.8)	899 (56.9)	121 (44.2)	
No	1628 (4.10)	717 (36.2)	77 (56.2)	681 (43.1)	153 (55.8)	
Hypertension						<0.001
Yes	1267 (31.9)	411 (20.7)	86 (62.8)	575 (36.4)	195 (71.2)	
No	2705 (68.1)	1570 (79.3)	51 (37.2)	1005 (63.6)	79 (28.8)	
Congestive heart failure						<0.001
Yes	73 (1.8)	13 (0.7)	4 (2.9)	31 (2.0)	25 (9.1)	
No	3899 (98.2)	1968 (99.3)	133 (97.1)	1549 (98.0)	249 (90.9)	
Coronary heart disease						<0.001
Yes	73 (1.8)	16 (0.8)	7 (5.1)	31 (2.0)	19 (6.9)	
No	3899 (98.2)	1965 (99.2)	130 (94.9)	1549 (98.0)	255 (93.1)	
Pulmonary emphysema						0.115
Yes	50 (1.3)	19 (1.0)	1 (0.7)	23 (1.5)	7 (2.6)	
No	3922 (98.7)	1962 (99.0)	136 (99.3)	1557 (98.5)	267 (97.4)	
Chronic bronchitis						<0.001
Yes	288 (7.3)	105 (5.3)	14 (10.2)	140 (8.9)	29 (10.6)	
No	3684 (92.7)	1876 (94.7)	123 (89.8)	1440 (91.1)	245 (89.4)	
Metformin use						<0.001
Yes	78 (2.0)	2 (0.1)	25 (18.2)	3 (0.2)	48 (17.5)	
No	3894 (98.0)	1979 (99.9)	112 (81.8)	1577 (99.8)	226 (82.5)	
HbA1c (%)	5.59±1.01	5.31±0.55	7.17±1.60	5.45±0.62	7.53±1.85	<0.001
CRP, mg/dL	0.50±0.74	0.43±0.70	0.57±0.60	0.55±0.78	0.66±0.74	<0.001
WBC, 1000 cells/µL	7.43±2.40	7.27±2.08	7.87±2.36	7.55±2.57	7.68±3.35	<0.001
NLR	2.20±1.13	2.15±1.09	2.11±0.91	2.29±1.19	2.08±1.27	0.001
PLR	142.3 ± 53.4	143.2 ± 53.1	133.0 ± 47.7	142.9 ± 51.6	136.0 ± 66.3	0.008
SIRI	1.18±0.83	1.14±0.79	1.16±0.75	1.26±0.88	1.09±0.79	<0.001
SII	632.3 ± 377.1	623.4 ± 372.4	611.0 ± 309.4	650.7 ± 370.9	602.0 ± 463.7	0.070

Note: Categorical variables were tested using Chi-Square tests.

Abbreviations: LUTS, Lower Urinary Tract Symptoms; DM, Diabetes Mellitus; BMI, Body Mass Index; WBC, White Blood Cell; NLR, Neutrophil-to-Lymphocyte Ratio; PLR, Platelet-to-Lymphocyte Ratio; SIRI, Systemic Inflammation Response Index; SII, Systemic Inflammation Index.

### Comparison of general condition among male participants

The clinical baseline data of male participants are compared in Table 2. The four groups consisted of 1478 (normal group, 77.7%), 246 (DM group, 12.9%), 136 (LUTS group, 7.2%), and 41 (diabetes‑associated LUTS group, 2.2%) individuals, respectively. Significant differences were observed among the four groups in terms of age, race, education level, BMI, smoking, alcohol use, hypertension, congestive heart failure, coronary heart disease, chronic obstructive pulmonary disease, chronic bronchitis, and metformin use. The male diabetes‑associated LUTS group had the highest proportions of individuals over 50-years-old, Mexican Americans, BMI ≥30 kg/m^2^, alcohol consumption, congestive heart failure, coronary heart disease, pulmonary emphysema, and chronic bronchitis, while the proportions of those with a college or above education and those who never smoked were the lowest.

**Table 2:** Clinical baseline characteristics of the 1901 male participants between 2005 and 2008.

Variables	Total patients	Normal	DM	LUTS	Diabetes‑associated LUTS	p-value
	n (%)	n (%)	n (%)	n (%)	n (%)	
Total patients	1898	1476 (77.8)	245 (12.9)	136 (7.2)	41 (2.2)	
Age, years						<0.001
≤ 50	722 (38.0)	631 (42.8)	50 (20.4)	35 (25.7)	6 (14.6)	
> 50	1176 (62.0)	845 (57.2)	195 (79.6)	101 (74.3)	35 (85.4)	
Race						0.036
Mexican Americans	365 (19.2)	275 (18.6)	57 (23.3)	23 (16.9)	10 (24.4)	
Other Hispanics	142 (7.5)	115 (7.8)	17 (6.9)	7 (5.1)	3 (7.3)	
Non-Hispanic whites	931 (49.0)	746 (50.5)	95 (38.8)	74 (54.4)	16 (39.0)	
Non-Hispanic blacks	394 (20.8)	285 (19.3)	69 (28.2)	29 (21.3)	11 (26.8)	
Other races	66 (3.5)	55 (3.7)	7 (2.9)	3 (2.2)	1 (2.4)	
Marital status						0.677
Married	1255 (66.1)	969 (65.7)	170 (69.4)	88 (64.7)	28 (68.3)	
Unmarried	643 (33.9)	507 (34.3)	75 (30.6)	48 (35.3)	13 (31.7)	
Educational level						<0.001
Less than high school	608 (32.0)	428 (29.0)	115 (46.9)	47 (34.6)	18 (43.9)	
High school	460 (24.2)	370 (25.1)	43 (17.6)	35 (25.7)	12 (29.3)	
College or above	830 (43.7)	678 (45.9)	87 (35.5)	54 (39.7)	11 (26.8)	
BMI, kg/m^2^						<0.001
< 25	449 (23.7)	366 (24.8)	37 (15.1)	40 (29.4)	6 (14.6)	
25‒29.9	800 (42.1)	644 (43.6)	88 (35.9)	54 (39.7)	14 (34.1)	
≥ 30	649 (34.2)	466 (31.6)	120 (49.0)	42 (30.9)	21 (51.2)	
Smoking						<0.001
Never	765 (40.3)	613 (41.5)	100 (40.8)	41 (30.1)	11 (26.8)	
Former	637 (33.6)	461 (31.2)	109 (44.5)	50 (36.8)	17 (41.5)	
Current	496 (26.1)	402 (27.2)	36 (14.7)	45 (33.1)	13 (31.7)	
Alcohol consumption						<0.001
Yes	348 (18.3)	241 (16.3)	64 (26.1)	31 (22.8)	12 (29.3)	
No	1550 (81.7)	1235 (83.7)	181 (73.9)	105 (77.2)	29 (70.7)	
Hypertension						<0.001
Yes	724 (38.1)	480 (32.5)	149 (60.8)	70 (51.5)	25 (61.0)	
No	1174 (61.9)	996 (67.5)	96 (39.2)	66 (48.5)	16 (39.0)	
Congestive heart failure						<0.001
Yes	81 (4.3)	34 (2.3)	29 (11.8)	10 (7.4)	8 (19.5)	
No	1817 (95.7)	1442 (97.7)	216 (88.2)	126 (92.6)	33 (80.5)	
Coronary heart disease						<0.001
Yes	121 (6.4)	71 (4.8)	32 (13.1)	11 (8.1)	7 (17.1)	
No	1777 (93.6)	1405 (95.2)	213 (86.9)	125 (91.9)	34 (82.9)	
Pulmonary emphysema						<0.001
Yes	50 (1.7)	25 (1.7)	14 (5.7)	7 (5.1)	4 (9.8)	
No	1848 (97.4)	1451 (98.3)	231 (94.3)	129 (94.9)	37 (90.2)	
Chronic bronchitis						<0.001
Yes	96 (5.1)	58 (3.9)	21 (8.6)	11 (8.1)	6 (14.6)	
No	1802 (94.9)	1418 (96.1)	224 (91.4)	125 (91.9)	35 (85.4)	
Metformin use						<0.001
Yes	49 (2.6)	1 (0.1)	43 (17.6)	0 (0.0)	5 (12.2)	
No	1849 (97.4)	1475 (99.9)	202 (82.4)	136 (100.0)	36 (87.8)	
HbA1c (%)	5.87±1.22	5.57±0.73	7.52±1.90	5.69±0.88	7.42±2.06	<0.001
CRP, mg/dL	0.41±0.81	0.39±0.71	0.43±0.69	0.63±1.67	0.64±0.74	0.023
WBC, 1000 cells/µL	7.19±2.15	7.11±2.00	7.43±2.24	7.28±2.02	8.43±5.06	<0.001
NLR	2.20±1.18	2.17±1.14	2.29±1.12	2.45±1.61	2.11±1.25	0.028
PLR	131.5 ± 51.2	132.2 ± 49.8	128.9 ± 52.0	134.3 ± 63.9	111.4 ± 46.1	0.058
SIRI	1.28±0.91	1.24±0.84	1.35±0.85	1.57±1.45	1.31±0.86	<0.001
SII	557.5 ± 341.5	553.1 ± 334.6	574.0 ± 339.2	596.0 ± 424.1	487.3 ± 285.3	0.268

Note: Categorical variables were tested using Chi-Square tests.

Abbreviations: LUTS, Lower Urinary Tract Symptoms; DM, Diabetes Mellitus; BMI, Body Mass Index; WBC, White Blood Cell; NLR, Neutrophil-to-Lymphocyte Ratio; PLR, Platelet-to-Lymphocyte Ratio; SIRI, Systemic Inflammation Response Index; SII, Systemic Inflammation Index.

### Comparison of inflammatory markers among the four groups

**Fig. 2:**
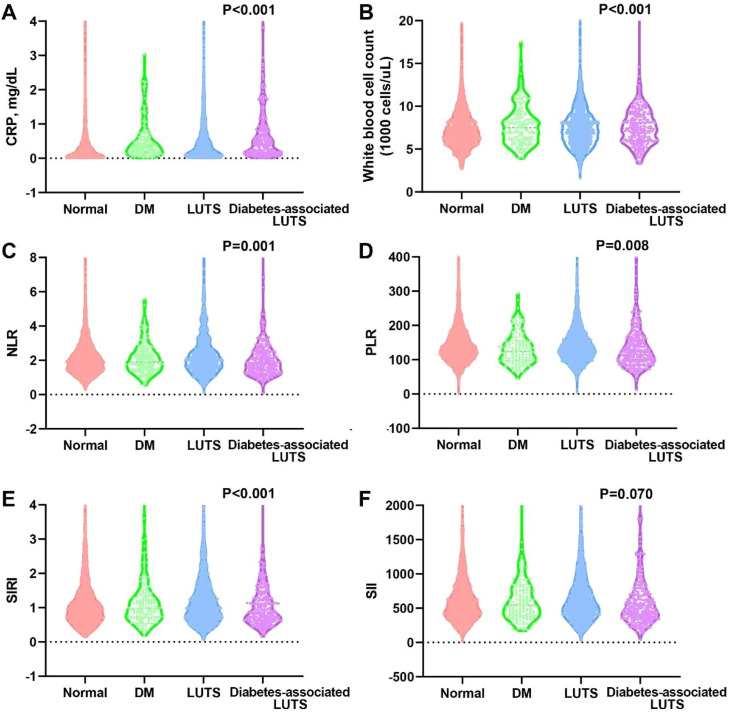
Inflammatory indicators in the four groups among female participants. (A) CRP; (B) WBC; (C) NLR; (D) PLR; (E) SIRI; (F) SII.

**Fig. 3:**
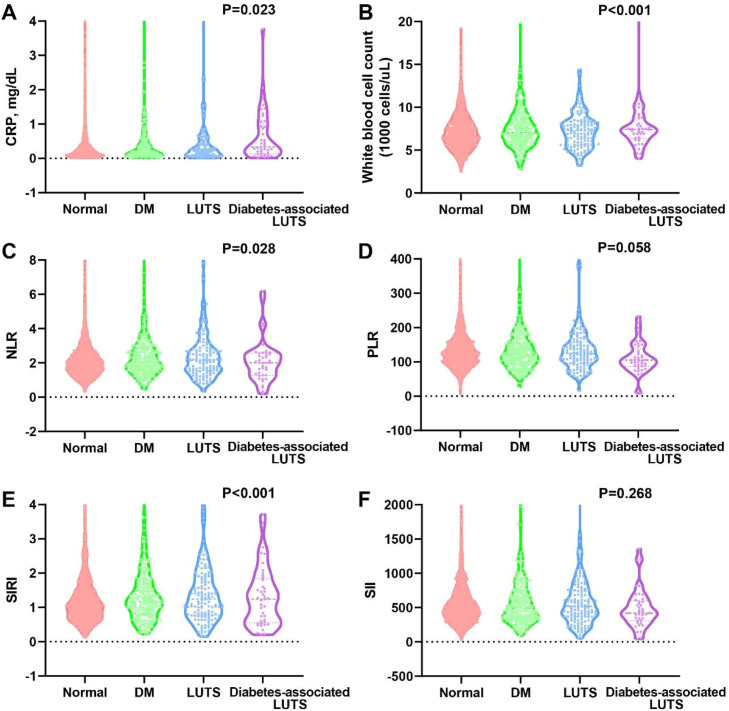
Inflammatory indicators in the four groups among male participants. (A) CRP; (B) WBC; (C) NLR; (D) PLR; (E) SIRI; (F) SII.

We also compared the expression levels of six different inflammatory markers across the four groups. In female participants, significant differences were observed in HbA1c, CRP, WBC, NLR, PLR, and SIRI among the four groups, while no significant difference was found in SII (Fig. 2). In male participants, significant differences were observed in HbA1c, CRP, WBC, NLR, and SIRI, while no significant differences were found in PLR and SII (Fig. 3). Additionally, in both male and female participants, the diabetes‑associated LUTS group exhibited the highest levels of CRP and WBC.

### WBC as an associated factor for diabetes‑associated LUTS

We subsequently used multivariate logistic regression to analyze the relationship between different inflammatory markers and diabetes‑associated LUTS. The results showed that, after adjusting for age, race, marital status, education level, BMI, smoking, alcohol use, hypertension, congestive heart failure, coronary heart disease, chronic obstructive pulmonary disease, chronic bronchitis, and metformin use, WBC was an associated factor for diabetes‑associated LUTS in both female and male participants. In female participants, high WBC increased the risk of diabetes‑associated LUTS by 10.7% (OR = 1.107, 95% CI 1.016‒1.205, *p* = 0.020), while in male participants, high WBC increased the risk of diabetes‑associated LUTS by 20.2% (OR=1.202, 95% CI 1.077‒1.342, *p* = 0.001) (Table 3).

**Table 3:** Multivariate logistical regression analysis of the relationship between inflammation indices and diabetes‑associated LUTS.

Variables	Adjusted OR (95% CI)	p-value
Female		
CRP	1.085 (0.883‒1.333)	0.439
WBC	1.107 (1.016‒1.205)	0.020
NLR	1.015 (0.876‒1.177)	0.841
PLR	0.998 (0.996‒1.001)	0.280
SIRI	1.035 (0.819‒1.309)	0.771
SII	1.000 (1.000‒1.000)	0.976
Male		
CRP	1.286 (0.910‒1.818)	0.154
WBC	1.202 (1.077‒1.342)	0.001
NLR	0.780 (0.537‒1.133)	0.192
PLR	0.989 (0.981‒0.998)	0.015
SIRI	0.888 (0.556‒1.418)	0.618
SII	0.999 (0.997‒1.000)	0.079

Note: LUTS, Lower Urinary Tract Symptoms; CRP, C-Reactive Protein; WBC, White Blood Cell Count; NLR, Neutrophil-to-Lymphocyte Ratio; PLR, Platelet-to-Lymphocyte Ratio; SIRI, Systemic Inflammation Response Index; SII, Systemic Inflammation Index.

## Discussion

DM is a metabolic disorder caused by various etiologies, characterized by chronic hyperglycemia due to insufficient insulin secretion and/or impaired utilization.^18^ DM is prone to trigger multiple complications, such as edema, severe hypertension, visual impairment or blindness, dry skin on the feet, and abdominal distension.^19^ Currently, the treatment of DM and its complications has become a global issue. In 2018, there were 34.2 million DM patients in the United States, and diabetes‑associated LUTS is one of the common complications in advanced DM patients.^20^^,^^21^ Typical symptoms of diabetes‑associated LUTS include decreased bladder sensation, increased capacity, difficulty in urination, and increased residual urine volume, severely affecting the quality of life of patients.

The etiology of diabetes‑associated LUTS is not fully understood, and researchers generally believe that it is related to the course of DM and the incidence and extent of peripheral neuropathy, among many other factors.^22^ Recent studies have shown that diabetes‑associated LUTS exhibits a chronic low-grade inflammatory response, which may be related to long-term hyperglycemia, induced polyuria, and oxidative stress.^23^ Insulin Resistance (IR) is a core feature of DM,^24^ and studies have reported the important role of insulin signaling in regulating bladder function, with disruption of the insulin signaling pathway leading to bladder dysfunction.^25^ Inflammatory responses play a crucial role in DM and its complications, with inflammatory factors and acute-phase reactants closely related to IR, such as Interleukin (IL)-1β, Nuclear Factor κB (NF-κB), IL-6, and Tumor Necrosis Factor-α (TNF-α).^26^ A persistent systemic inflammatory state can lead to the release of more pro-inflammatory factors through circulating inflammatory mediators (such as white blood cells, neutrophils, lymphocytes) and locally infiltrating immune cells.^27^ Studies have reported that inhibiting inflammatory responses can improve bladder dysfunction caused by DM.^28^

WBCs are used clinically as indicators of infection and systemic inflammatory status.^29^ Numerous studies have shown that WBC count is positively correlated with all-cause mortality and mortality rates in patients with coronary heart disease, ischemic stroke, and cancer.^30^ Brown et al.^31^ found that a higher WBC count is an independent predictor of mortality in patients with coronary heart disease. Peripheral blood WBC count can serve as a macroscopic indicator of the immune system and the degree of inflammation, reflecting the severity of the condition.^32^ WBCs can lead to elevated levels of pro-inflammatory cytokines, chemokines, and reactive oxygen species in the blood.^33^ Pro-inflammatory cytokines TNF-α and IL-1β can directly act on bladder nerves, activating receptors on neurons and triggering intracellular apoptotic signaling pathways, resulting in neuronal cell death or axonal damage.^34^ Furthermore, factors like TGF-β1 activate fibroblasts within the bladder interstitium, inducing their differentiation into highly contractile and synthetic myofibroblasts, leading to fibrosis of the bladder detrusor muscle.^35^ In this study, the results showed that diabetes‑associated LUTS patients have higher WBC counts, and WBC count is an associated factor for diabetes‑associated LUTS patients.

The present study also has some limitations. Firstly, the NHANES database is a retrospective cross-sectional study, and prospective studies are needed. Secondly, the diagnosis of diabetes‑associated LUTS was based on self-reported lower urinary tract symptoms from the NHANES questionnaire, which lacks clinical validation and introduces misclassification bias. Furthermore, this study assessed “LUTS in diabetic patients” rather than urodynamically confirmed cystopathy. Additionally, the causal relationship between WBC count and diabetes‑associated LUTS cannot be established, and longitudinal studies are required to assess their temporal relationship.

## Conclusion

WBC count is an associated factor for diabetes‑associated LUTS patients, and incorporating WBC count into clinical assessments can help identify diabetes‑associated LUTS patients.

## Abbreviations

LUTS, Lower Urinary Tract Symptoms; NHANES, National Health and Nutrition Examination Survey; CRP, C-Reactive Protein; DM, Diabetes Mellitus; BMI, Body Mass Index; WBC, White Blood Cell; NLR, Neutrophil-to-Lymphocyte Ratio; PLR, Platelet-to-Lymphocyte Ratio; SIRI, Systemic Inflammation Response Index; SII, Systemic Inflammation Index; OR, Odds Ratios; CI, Confidence Intervals; ROS, Reactive Oxygen Species; NCHS, National Center for Health Statistics; IR, Insulin Resistance; NF-κB, Nuclear Factor-Kappa B; TNF-α, Tumor Necrosis Factor-α.

## Data availability statement

The NHANES data used in this work are publicly available. All raw data are available on the NHANES website (https://wwwn.cdc.gov/nchs/nhanes/default.aspx). The datasets used and analyzed during the current study are available from the corresponding author on reasonable request.

## Statement of ethics

The NHANES study protocol was approved by the NCHS Research Ethics Review Board, and all participants provided written informed consent. This study used previously collected deidentified data, which was deemed exempt from review by the Ethics Committee of the Affiliated Zhongda Hospital of Southeast University (2024ZDSYLL458-P01).

## Authors’ contributions

Conception and design: NL and NC; Administrative support: NL and DZ; Provision of study materials or patients: NL and DZ; Collection and assembly of data: NL and DZ; Data analysis and interpretation: NL, DZ and NC; Manuscript writing: NL. Final approval of manuscript: All authors.

## Funding

This work was supported by the Opening Foundation of Key Laboratory (JSHD202311) and Jiangsu Province Capability Improvement Project through Science, Technology and Education (ZDXYS202210).

## Declaration of competing interest

The authors declare no conflicts of interest.

## Appendix Supplementary materials

Image, application 1
