# Supplementary material for: Risk predictive value of white blood cell count in patients with diabetes‑associated lower urinary tract symptoms: A population-based study
Source: Clinics (Sao Paulo). 2026 Apr 17;81:100928. doi: 10.1016/j.clinsp.2026.100928 (PMC13098451; doi:10.1016/j.clinsp.2026.100928)

**CLINICS-D-25-00508_Supplementary Material**

**Figure S1** Dose-response curves and receiver operating characteristic curves between WBC count and diabetes‑associated LUTS. (A) Dose-response curves between WBC count and diabetes‑associated LUTS among female participants; (B) Dose-response curves between WBC count and diabetes‑associated LUTS among male participants; (C) Receiver operating characteristic curves between WBC count and diabetes‑associated LUTS among female participants; (D) Receiver operating characteristic curves between WBC count and diabetes‑associated LUTS among male participants.


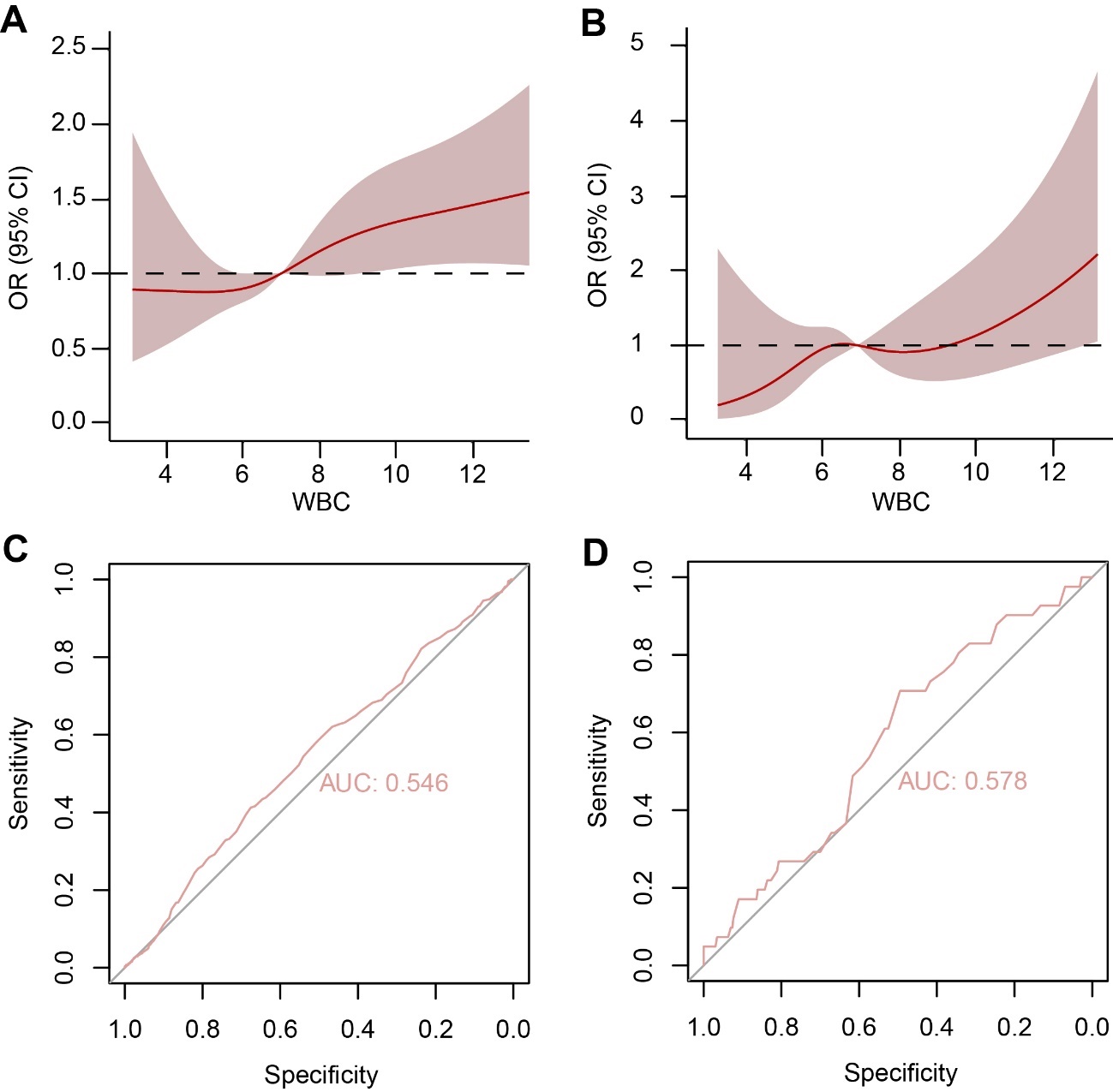

Supplement: Supplementary file 1 [file mmc1.docx]
